# Potential for improvement of docetaxel-based chemotherapy: a pharmacological review

**DOI:** 10.1038/sj.bjc.6602698

**Published:** 2005-07-12

**Authors:** F K Engels, A Sparreboom, R A A Mathot, J Verweij

**Affiliations:** 1 Department of Medical Oncology, Erasmus MC - Daniel den Hoed Cancer Center Rotterdam, Groene Hilledijk 301, 3075 EA Rotterdam, The Netherlands; 2 Clinical Pharmacology Research Core, Medical Oncology Clinical Research Unit, National Cancer Institute, Bethesda, MD; 3 Department of Hospital Pharmacy and Clinical Pharmacology, Erasmus MC, Rotterdam, The Netherlands

**Keywords:** docetaxel, taxotere, taxanes, pharmacology

## Abstract

Since the introduction of docetaxel, research has focused on various approaches to overcome treatment limitations and improve outcome. This review discusses the pharmacological attempts at treatment optimisation, which include reducing interindividual pharmacokinetic and pharmacodynamic variability, optimising schedule, route of administration, reversing drug resistance and the development of structurally related second-generation taxanes.

The anticancer drug docetaxel (Taxotere®) is approved for the treatment of patients with locally advanced or metastatic breast or non-small-cell lung cancer and androgen-independent metastatic prostate cancer. The recommended dose ranges from 60 to 100 mg m^− 2^ given as a 1-h intravenous (i.v.) infusion once every 3 weeks. An important limitation associated with docetaxel use is the unpredictable interindividual variability in efficacy and toxicity. Since its clinical introduction, attempts to improve docetaxel treatment have covered various areas: reducing the interindividual pharmacokinetic (PK) and pharmacodynamic (PD) variability, optimising schedule, route of administration and drug formulation, and reversing drug resistance. This review will discuss pharmacological strategies aimed to overcome the limitations of docetaxel therapy.

## ALTERNATIVE SCHEDULES

When treated at a dose of 100 mg m^− 2^ once every 3 weeks, grade 4 neutropenia and febrile neutropenia occur in 75%, respectively 11%, of patients; a dose of 75 mg m^− 2^ only moderately reduces this incidence (http://www.taxotere.com). For patients with a poor performance status (PS), multiple comorbidities, decreased haematological reserves, a history of extensive pretreatment and severe toxicity, elderly patients and for patients for whom treatment is palliative, a less toxic schedule seemed desirable. Therefore, a schedule involving weekly administration was developed. Numerous trials have evaluated this schedule; however, due to considerably different study populations and small sample sizes, comparisons of weekly *vs* 3-weekly efficacy were difficult. Recent randomised trials although demonstrate, for the approved indications, that the efficacy of weekly docetaxel is comparable to 3-weekly treatment (Engels and Verweij, 2005), the toxicity profiles are, however, distinctly different. With weekly docetaxel, acute toxicities, in particular myelosuppression, are mild and never dose limiting. In contrast, cumulative side effects are much more prominent. The most common and dose-limiting toxicity is fatigue/asthenia. These side effects can only be managed by reducing the dose or by shortening the schedule to 2–3 consecutive weekly infusions, followed by a 1-week rest interval. Other cumulative toxicities include alopecia, excessive tearing and nail disorders. Although the latter two side effects are usually mild, they are persistent, can lead to treatment discontinuation and have a substantial negative impact on a patient's quality of life. Given the similar efficacy observed for the two schedules and the remarks on toxicity, it is reasonable to conclude that, at this point, 3-weekly docetaxel is still the standard and most convenient schedule. Treatment with weekly docetaxel should only be considered as an alternative for specific patient populations.

## PK OPTIMISATION

The PK of total docetaxel are linear and independent of schedule. Nonetheless, there is a large interpatient variability in exposure (AUC) and drug clearance (Bruno *et al*, 1998; Hirth *et al*, 2000; Rudek *et al*, 2004; Baker *et al*, 2005; ten Tije *et al*, 2005). In a large population, PK/PD analysis variability in efficacy and toxicity was associated with variability in PK (Bruno *et al*, 1998); a 50% decrease in docetaxel clearance increased the odds of developing grade 4 neutropenia and febrile neutropenia 4.3-fold, respectively 3.0-fold. Subsequent studies have therefore focused on identifying factors, which most affect PK variability. Ultimately, reducing interpatient exposure variability should improve the risk–benefit ratio of docetaxel therapy.

Initially, the main predictors of total docetaxel clearance (variability) were body surface area (BSA), *α*1-acid glycoprotein (AAG), hepatic function (elevated alkaline phosphatase (ALKPH) and transaminases levels) and age (Bruno *et al*, 1996). More recently (hepatic), cytochrome *P*450 isozyme 3A4 (CYP3A4) activity was also included (Hirth *et al*, 2000). The relevance of all these predictors to docetaxel dose optimisation has been further (re-) evaluated.

Normalisation of clearance for BSA reduces interindividual variability marginally (<2%), thus questioning the clinical relevance of BSA-based dosing (Rudek *et al*, 2004). Clearance was, however, significantly higher by 33% (*P*=0.0029) for patients with BSA values >2.00 m^2^ compared to values <1.71 m^2^. Flat dosing, possibly differentiating for extremes of BSA (>2.00 m^2^), may be easier and just as precise and should be investigated prospectively.

In general, the unbound (i.e. free) fraction of any drug is pharmacologically active. In serum, docetaxel is extensively bound to albumin, lipoproteins and AAG; indeed, the latter is the main determinant of docetaxel serum binding variability. Furthermore, AAG levels in cancer patients vary at least four-fold (Bruno *et al*, 1998; Loos *et al*, 2003; Baker *et al*, 2005). High AAG levels have been associated with a decrease in the unbound fraction of docetaxel *in vitro*, and with reduced total docetaxel clearance (Bruno *et al*, 1996; Loos *et al*, 2003) and lower response rate in man (Bruno *et al*, 1998). Yet, dosing recommendations based on individual AAG levels are not available. The formulation vehicle polysorbate 80, although rapidly degraded by serum esterases, also influences docetaxel protein binding, increasing the unbound drug fraction by on average 16–24% at peak polysorbate 80 concentrations (Loos *et al*, 2003; Baker *et al*, 2005). Furthermore, higher polysorbate 80 exposure resulted in lower unbound docetaxel clearance. Most importantly, haematological toxicity is highly correlated with systemic exposure to unbound docetaxel (Baker *et al*, 2005). Thus, measuring unbound docetaxel concentrations should be considered in future PK/PD studies.

Mild hepatic impairment (total bilirubin <1.5 × ULN, transaminases ⩾1.5 to ⩽3.5 × ULN concurrent with ALKPH ⩾2.5 to ⩽5 × ULN) decreases total docetaxel clearance by 27% (Bruno *et al*, 1998), and moderate (total bilirubin ⩾1.5 to <3.0 × ULN with any transaminase and ALKPH elevations) to severe impairment (total bilirubin ⩾3.0 × ULN with any transaminase and ALKPH elevations) does so by 50%. This should obviously have consequences, yet one is only warned that docetaxel should *generally* not be given to patients with total bilirubin >ULN, or with transaminases >1.5 × ULN concomitant with ALKPH >2.5 × ULN (www.taxotere.com). Dose adjustments therefore are left to the discretion of the physician, while they seem required.

Elderly age (⩾65 years) does not alter total docetaxel PK (ten Tije *et al*, 2005). Interestingly, the elderly patients' serum AAG levels were significantly (*P*⩽0.04), albeit only slightly lower. Decreased AAG levels contribute to higher exposure to unbound docetaxel resulting in more myelosuppression (Baker *et al*, 2005). Jointly with the increased susceptibility for myelosuppression due to a functional decline in haematological reserves at ageing, this could be a concern. Yet, toxicity did not occur more frequently in the elderly. Thus, age-related dose recommendations should predominantly be based on individual PS and comorbidity.

Docetaxel is primarily metabolised by (hepatic and intestinal) CYP3A, in particular by isoforms CYP3A4 and CYP3A5, the latter of which has a 10-fold lower affinity for docetaxel. Docetaxel metabolites are substantially less active than the parent drug; hence, CYP3A-mediated metabolism is the major route of inactivation. CYP3A activity in adults, and in patients treated with docetaxel, varies largely between individuals. This is believed to depend on environmental (CYP3A modulation), physiological (hepatic impairment) and genetic (CYP3A polymorphism) factors. Pretreatment CYP3A phenotyping has been suggested as a tool to individualise docetaxel dosing (Rivory *et al*, 2000; Goh *et al*, 2002; Puisset *et al*, 2004; Yamamoto *et al*, 2005). At present, the midazolam hydroxylation test and the erythromycin breath test (ERMBT) are the most widely applied phenotyping strategies, albeit that both have their limitations. The ERMBT did not consistently correlate with results from other CYP3A phenotypic probes (Chiou *et al*, 2001) and may have limited value for CYP3A phenotyping of docetaxel patients as erythromycin is preferentially metabolised by CYP3A4, whereas docetaxel is metabolised by CYP3A4 *and* CYP3A5. Yet, when compared to other variables (ALKPH, alanine aminotransferase and AAG), the ERMBT was the best single predictor of docetaxel clearance (Hirth *et al*, 2000) and probe specificity issues are relevant only in individuals expressing significant CYP3A5 levels, which is rarely the case in Caucasians. In contrast, African-Americans have much higher CYP3A5 expression and in these patients midazolam, metabolised by both CYP3A4 and CYP3A5, may be more suitable. Clinical trials correlating phenotyping results to docetaxel PK demonstrate that midazolam, erythromycin and dexamethasone are predictors of docetaxel clearance (Hirth *et al*, 2000; Goh *et al*, 2002; Puisset *et al*, 2004). As dexamethasone is routinely used as premedication, it may be more attractive as a probe drug than midazolam or erythromycin. Recently, individualised dosing based on the 24-h urinary metabolite of exogenous cortisol as phenotypic CYP3A probe was evaluated (Yamamoto *et al*, 2005). Individualised phenotypic dosing significantly reduced the interindividual PK variability compared to BSA-based dosing. Further larger studies, preferably comparing phenotyping strategies, are required to assess which probe is the best predictor of CYP3A activity. Nonetheless, phenotyping techniques have practical disadvantages (i.e. 24-h urine collection, radioisotope administration) that, may limit their applicability in common oncology practice.

The involvement of CYP3A in docetaxel elimination renders the drug potentially subject to a host of enzyme-mediated PK drug interactions with conventional drugs, complementary and alternative medicine and food constituents that interfere with CYP3A function or expression. Docetaxel has a narrow therapeutic window. Therefore, the risk of a PK interaction resulting in under- or overexposure, thereby modifying treatment outcome, is high. For several coadministered cytotoxic agents, PK interactions with docetaxel are known and have led to dose or schedule recommendations. Interestingly, for the potent CYP3A inhibitor ketoconazole interaction data are inconsistent. Both trials observed large interindividual variability in the reduction of docetaxel clearance (Van Veldhuizen *et al*, 2003; Engels *et al*, 2004). Yet, in one this was highly significant (Engels *et al*, 2004) whereas in the other, although docetaxel clearance decreased 2–4-fold in 25% of the patients, thus increasing the risk for severe neutropenia, it was not (Van Veldhuizen *et al*, 2003). Efforts to reduce the interindividual PK variability through inhibition of CYP3A by ketoconazole have not been successful. No clinically relevant PK interaction has been observed between dexamethasone, a possible CYP3A inducer, and docetaxel (Hirth *et al*, 2000; Goh *et al*, 2002). Thus, there is no reason to abandon routine dexamethasone premedication. Clearly, the degree to which a PK interaction is clinically relevant, and requires an intervention depends upon the CYP3A-inducing or -inhibiting properties of the coadministered agent. Since specific dose adjustment recommendations are not available, concomitant administration of potent CYP3A-modulating comedication should generally be avoided.

Docetaxel is also a substrate for the ATP-binding cassette transmembrane transporter protein ABCB1 (P-glycoprotein (P-gp); MDR-1). ABCB1 is expressed in tumours and in normal tissues including the blood–brain–barrier (BBB), biliary tract and intestinal epithelium. Although ABCB1 plays a (major) role in the intestinal absorption and biliary excretion of *orally* administered substrates, its influence on the plasma PK of *i.v.* administered drugs, including docetaxel, is minimal to absent (van Zuylen *et al*, 2000). ABCB1 inhibition does, however, significantly influence the faecal disposition of docetaxel, reducing the amount of excreted unchanged drug (approximately 18-fold) without affecting plasma PK (van Zuylen *et al*, 2000), indicating that the effects of ABCB1 modulation on docetaxel PK cannot be evaluated when analysing only plasma.

Monitoring plasma levels and PK-guided dose adjustments is referred to as therapeutic drug monitoring (TDM). At present, the use of TDM in oncology is limited. A prerequisite for TDM is that *intra*individual PK variability is less than *inter*individual PK variability, which is the case for docetaxel. For reasons of patient convenience and practicality, validated limited sampling strategies (LDS), requiring only two to four samples to characterise an individual PK profile (Bruno *et al*, 1996, 1998), should be used. LDS used in combination with a population PK model and Bayesian analysis allows individual PK parameters to be estimated with adequate precision while sampling and dosing times remain flexible. TDM could become an interesting strategy to docetaxel dose individualisation provided a target concentration or exposure profile can be defined. It should be noted, however, that obtaining two to four samples from outpatients requires adequate planning and a good collaboration between pharmacy, outpatient clinic and the prescribing oncologist to assure that sampling can be completed within the service hours of an oncology day unit.

## REVERSAL OF DRUG RESISTANCE

The most extensively studied mechanism of acquired or intrinsic resistance to taxanes is the overexpression of ABCB1. Numerous (pre)clinical investigations have evaluated coadministration of ABCB1 (P-gp) modulators (e.g. verapamil, cyclosporin A (CSA), valspodar), aiming to restore or enhance sensitivity to chemotherapy. However, the results were largely disappointing. To overcome the limitations of these first- and second-generation modulators (unacceptable toxicity and unpredictable PK interactions), highly specific and potent third-generation ABCB1 modulators, lacking interference with the plasma PK of cytotoxics, were developed.

In phase I trials, oral and i.v. R101933 (laniquidar) administered in combination with docetaxel inhibited ABCB1 both in an *ex vivo* assay and *in vivo* (indicated by intestinal P-gp inhibition), and docetaxel plasma PK was not altered (van Zuylen *et al*, 2000, 2002). However, phase II studies were negative. Similar disappointing efficacy results were obtained in clinical trials of XR9576 (tariquidar) and docetaxel, and at present, there are no plans for further clinical development (www.qltinc.com). A phase I trial of docetaxel in combination with the orally administered agent LY335979 (zosuquidar) showed no PK interaction (Fracasso *et al*, 2004). The limited cerebrospinal fluid penetration of docetaxel is also assumed to be due to ABCB1-mediated drug efflux and restricts treatment of brain tumours with docetaxel. Preclinical investigations with GF120918 (elacridar) suggested increased docetaxel brain concentrations without any effect on plasma PK (Kemper *et al*, 2004), but a phase I trial reported increased systemic exposure to docetaxel and reduced clearance (Lokiec *et al*, 2003). This interaction will likely limit further clinical development.

Docetaxel is also a substrate of CYP1B1, a cytochrome isozyme not detected in human liver but (over)expressed in various tumours. *In vitro* docetaxel cytotoxicity in cells transfected with human CYP1B1 was decreased (McFadyen *et al*, 2001), but CYP1B1-mediated docetaxel metabolism was not affected (Bournique and Lemarie, 2002). Meanwhile, the ability of CYP1B1 inhibitors to increase the cytotoxic effect of docetaxel has recently been demonstrated *in vitro*. Clinical studies have not yet been performed and the functional role of intratumoral CYP1B1 (-mediated resistance) on docetaxel cytotoxicity remains to be elucidated.

Clearly, the ultimate (multi)drug resistance reversal agent is not (yet) available. Moreover, one should realise that modulating one resistance mechanism will not yield important antitumour benefit, given the large number of resistance mechanisms in human tumour tissue.

## ALTERNATIVE ROUTES OF ADMINISTRATION – *ORAL ADMINISTRATION*

*In vitro* increasing the duration of taxane exposure above a threshold level is more important than achieving high peak concentrations. Clinically, the duration of exposure to plasma levels greater than 0.080 *μ*g/ml indeed predicted response (Bruno *et al*, 1998). Oral docetaxel treatment would be a patient-convenient way to achieve long-term drug exposure. However, development of a suitable oral formulation has been impeded by low (<10%) and highly variable oral bioavailability, due to the discussed extensive CYP3A-mediated first-pass metabolism and, to a lesser degree, to affinity for outward-directed transport by ABCB1 in the gastrointestinal tract. Modulating these elimination routes has therefore been a focus of research.

In wild-type mice, exposure to orally administered docetaxel was six-fold lower compared to *Abcb1a/1b* knockout mice (Bardelmeijer *et al*, 2002). More importantly, the relative bioavailability increased from 4 to 183% by coadministration of the potent CYP3A (and poor ABCB1) inhibitor ritonavir, increasing systemic exposure 50-fold. Subsequently, a small PK study, in which patients were given oral docetaxel (75 mg m^− 2^) with or without the ABCB1 and CYP3A inhibitor CsA, confirmed the observation (Malingre *et al*, 2001). In the presence of CsA, systemic exposure increased approximately seven-fold (from 0.37±0.33 to 2.71±1.81 mg h^− 1^ l^− 1^). When given 100 mg m^− 2^ docetaxel *i.v.* (without CsA), the resulting systemic exposure was 4.27±2.26 mg h^− 1^ l^− 1^. Adjusted for the difference in dose, exposure following *oral* administration *with* concomitant CsA does not greatly differ from exposure after *i.v.* administration *without* CsA. The investigators performed a phase II trial with weekly oral docetaxel (100 mg) in combination with CsA (Kruijtzer *et al*, 2001). Interpatient PK variability, haematological toxicity and antitumour activity seem to be in the same range as for intravenous docetaxel. Oral docetaxel (100 mg) was also combined with OC144-093, a potent and selective oral ABCB1 inhibitor, and compared to 100 mg i.v. docetaxel (Kuppens *et al*, 2005). The relative oral bioavailability of docetaxel was 26±8%, lower than previously observed after CsA coadministration and systemic exposure after i.v. docetaxel was administered three-fold higher compared to the oral application, despite the ABCB1 modulation. This indicating that CYP3A-mediated (first-pass) metabolism is the crucial process involved in the poor oral bioavailability of docetaxel.

Notwithstanding the fact that the oral bioavailability of docetaxel can be increased through pharmacologic modulation, the development of second-generation oral taxanes is likely to prevail.

## SECOND-GENERATION DOCETAXEL-BASED TAXANES

Lately, structure-activity relationship studies have focused on identifying novel structurally related docetaxel analogues with increased cytotoxicity in resistant tumours, increased penetration across the BBB, decreased toxicity, oral bioavailability and higher water solubility, the latter facilitating drug formulation. Chemical modification of the core structure of docetaxel has resulted in docetaxel-based second-generation taxanes, which are in different phases of clinical development (Table 1).

Docetaxel is synthesised from 10-deacetylbaccatin III, a noncytotoxic precursor derived from the European yew tree. Research initially focused on modifications of this compound and yielded XRP9881 (RPR109881A) and XRP6258 (RPR116258A or TXD258). Both agents have comparable mechanism of action to docetaxel, and in tumour models sensitive to docetaxel, cytotoxic activity was similar to docetaxel (http://www.AventisOncology.com). Importantly, *in vitro* these agents are characterised by potent growth inhibitory activity in moderately and highly docetaxel-resistant cell lines, most probably based upon a substantially lower affinity for ABCB1. Furthermore, glioblastoma models proved to be sensitive to these agents, suggesting penetration of the BBB. Phase I trials and early phase II studies with XRP9881 in metastatic breast cancer patients suggest adequate activity (Kurata *et al*, 2000). A differentiating feature of XRP6258 is its antitumour activity following oral administration, yet initial development is as intravenous administration. Both agents demonstrate marked interpatient variability in drug clearance, similar to docetaxel. Short-lasting and manageable neutropenia, fatigue and diarrhoea are the dose-limiting toxicities.

Several cytotoxic analogues derived from 14-*β*-hydroxy-10-deacetylbaccatin III, a natural compound closely related to the core structure of docetaxel, have been evaluated. The most interesting is ortataxel (IDN5109, BAY 59-8862). Ortataxel has adequate oral bioavailability and can modulate the function of various ABC transporter proteins, including ABCB1, MRP and BCRP (Minderman *et al*, 2004). The drug is not active in renal cancer patients, and phase II studies in taxane-resistant metastatic breast cancer and NCSLC patients are ongoing. MAC-321 or TL00139 exhibits a similar mechanism of cytotoxic activity as docetaxel (Sampath *et al*, 2003), is highly effective both orally and intravenously administered and currently under investigation involving both administration routes. DJ-927 is a novel semisynthetic taxane with high water solubility, lack of neurotoxicity, good oral bioavailability and superior antitumour activity compared to docetaxel in *in vitro* and *in vivo* models (Shionoya *et al*, 2003). Preliminary results of a phase I trial of orally administered DJ-927 suggest that the agent may have favourable toxicological and pharmacological properties.

## CONCLUSION

Continued research has offered us new and complementary insights on various aspects of docetaxel treatment, and yet, dose and schedule are still based on initial recommendations. Although this may sound disappointing, important steps forward have been made (Table 2) and research is ongoing. Besides the discussed areas of treatment optimisation, future investigations will focus on further development of preclinically promising alternative formulations, on pharmacogenomic-based treatment optimisation and on pharmacogenetic-based dose individualisation strategies. However, given the large, ethnically diverse population studies required, introduction of the latter two strategies is not expected in the foreseeable future. On shorter term, it is likely that TDM will be explored as it provides a potential tool for rapidly achievable treatment optimisation.

## Figures and Tables

**Table 1:** Second-generation taxanes, structurally related to docetaxel

**Drug**	**Nature of derivative**	**Cytotoxicity^a^**	**Cytotoxicity^b^**	**Developmental phase**	**Administration route(s) and recommended dose**	**Firm**
XRP9881	10-DAB	Similar	Superior	Phase II	i.v. 90 mg m^− 2^, q 3 wks	Aventis Pharma
XRP6258	10-DAB	Similar	Superior	Phase I	i.v. 30 mg m^− 2^, q 3 wks also orally active	Aventis Pharma
Ortataxel	14-*β*-hydroxy-DAB	Similar	Superior^c^	Phase II	i.v. 75 mg m^− 2^, q 3 wks also orally active	Bayer/Indena
MAC-321	10-deacetyl-7-propanoyl baccatin	Similar	Superior^d^	Phase II	i.v. 40 mg m^− 2^, q 3 wksOral 60 mg m^− 2^, q 3 wks	Wyeth-Ayerst
DJ-927	7-deoxy-9-*β*-dihydro-9,10,*O*-acetal taxane	Similar	Superior^e^	Phase I	Oral; 27 mg m^− 2^, q 3 wks	Daiichi Pharmaceuticals

a Compared to docetaxel-sensitive cell lines and human xenografts.

b Compared to docetaxel (highly and moderately) resistant cell lines and human xenografts (over)expressing *Abcb*-1.

c More potent (20–30-fold) in human breast and colon cancer cell lines.

d Drug resistance in KBV1 cells: eight-fold lower for MAC-321.

e More potent (40–50-fold) in PC-6/VCR29-1 cell lines.

DAB=deacetyl baccatin III; wks=weeks; i.v.=intravenous.

**Table 2:** Investigated areas of improvement of docetaxel-based chemotherapy

**Area of improvement**	**Outcome**
Weekly schedules	Alternative for patients at high risk for myelotoxic complications
PK	Interindividual variability can be decreased by phenotypic individualised dosing
	Most predictive phenotyping probe controversial
	Practical disadvantages of phenotyping in oncology practice
	TDM requires investigation
Reversal of resistance	ABCB1-modulating agents insufficiently reverse (multi)drug resistance due to multiple resistance mechanisms
Oral administration	Oral administration feasible upon pharmacologic modulation
	Second-generation oral taxanes likely to prevail
Second-generation taxanes	In clinical phase I/II development; also oral drugs
Alternative formulations	Alternative formulations in preclinical phase
	Introduction not foreseen in near future
Pharmacogenomics and pharmacogenetics	Sufficiently powered trials necessary to determine clinical relevance

PK=pharmacokinetics; TDM=therapeutic drug monitoring.
